# A novel zoonotic *Anaplasma* species is prevalent in small ruminants: potential public health implications

**DOI:** 10.1186/s13071-017-2182-9

**Published:** 2017-05-30

**Authors:** Jifei Yang, Zhijie Liu, Qingli Niu, Junlong Liu, Rong Han, Guiquan Guan, Muhammad Adeel Hassan, Guangyuan Liu, Jianxun Luo, Hong Yin

**Affiliations:** 1 State Key Laboratory of Veterinary Etiological Biology, Key Laboratory of Veterinary Parasitology of Gansu Province, Lanzhou Veterinary Research Institute, Chinese Academy of Agricultural Science, Xujiaping 1, Lanzhou, Gansu 730046 People’s Republic of China; 2 Jiangsu Co-innovation Center for Prevention and Control of Important Animal Infectious Diseases and Zoonoses, Yangzhou, 225009 People’s Republic of China

**Keywords:** *Anaplasma capra*, Prevalence, 16S rRNA gene, *gltA* gene, *groEL* gene, *msp4* gene, Anaplasmosis, Public health

## Abstract

**Background:**

Tick-borne diseases currently represent an important issue for global health. A number of emerging tick-transmitted microbes continue to be discovered, and some of these are already identified as the cause of human infections. Over the past two decades, *Anaplasma phagocytophilum* is considered to be mainly responsible for human anaplasmosis. However, a novel zoonotic pathogen provisionally named “*Anaplasma capra*” has recently been identified in China. In this study, we did an active surveillance of *A. capra* in goats and sheep in different geographical regions of China.

**Methods:**

The presence of *A. capra* was determined by nested PCR in 547 blood samples collected from goats and sheep from 24 counties distributed in 12 provinces in China. The molecular characterization of *A. capra* isolates in sheep and goats was achieved based on four conventional genetic markers (16S rRNA, *gltA*, *groEL* and *msp4* genes).

**Results:**

*Anaplasma capra* was identified in 75 of 547 animals, with an overall prevalence of 13.7%. The infection rates in the survey sites ranged from 0 to 78.6%, and were significantly different (*P* < 0.01). Phylogenetic analysis revealed that the isolates obtained from goats, sheep, *Ixodes persulcatus* ticks and humans create a separate clade within the genus *Anaplasma* and distinct from other recognized *Anaplasma* species. These findings indicated that these *A. capra* isolates possess the same molecular characteristics, suggesting that this organism could be a substantial health threat to both animals and humans.

**Conclusions:**

*Anaplasma capra* is an emerging tick-transmitted zoonotic pathogen. This novel *Anaplasna* species is widespread across China with an overall prevalence of 13.7% in goats and sheep with isolates indistinguishable from those found in humans. These findings warrant increased public health awareness for human anaplasmosis.

## Background

Tick-borne diseases are currently becoming an important threat for public health worldwide [1]. With the development of molecular techniques, an increasing number of novel species and genetic variants of tick-borne pathogens are being detected in ticks and/or animals, some of which have been recognized as human pathogens [2]. The majority of emerging tick-borne infections was discovered during the last 20 years [3]. To date, 33 emerging pathogens associated with tick vectors have been identified in China, including the species in the complex *Borrelia burgdorferi* (*sensu lato*) (*s.l*.), spotted fever group rickettsiae, *Anaplasma*, *Babesia* and severe fever with thrombocytopenia syndrome virus (SFTSV) [2]. The identification of novel tick-borne pathogens will facilitate development of ecological and public health measures to control and manage these health threats.

In 2015, a new tick-transmitted *Anaplasma* species provisionally named “*Anaplasma capra*” was identified in goats and ticks in northern China [4]. This organism was considered to be an emerging human pathogen and is phylogenetically distinct from other established *Anaplasma* species [4]. Twenty-eight human cases caused by *A. capra* have been reported in Heilongjiang Province in northeast China, and the pathogen was isolated from three patients [4]. The disease caused by *A. capra* presents non-specific symptoms with fever, headache, malaise, rash, eschar and chills [4]; these common signs would be very difficult to distinguish clinically from other tick-borne illnesses. Although *A. capra* was first recognized in goats (*Capra aegagrus hircus*) in northeastern China, the 16S rRNA sequences of *A. capra* have previously been detected in goats from south and southwest China [5, 6], in deer and also serows from Japan [7, 8], implying that *A. capra* may be widely distributed in China and elsewhere. The objective of this study was to determine the occurrence, prevalence and molecular characterization of *A. capra* in goats and sheep in different geographic regions of China.

## Methods

### Study sites and collection of specimens

From 2011 to 2015, samples were collected between March and September, to provide a total of 547 EDTA-anticoagulated blood samples from asymptomatic sheep and goats in rural villages from 24 counties in 12 provinces of China. Two to three flocks were selected for sampling in each county. DNA was extracted from 300 μl of whole blood using the Gentra Puregene Blood kit (Qiagen, Beijing, China) according to the manufacturer’s instructions.

### PCR reactions

DNA samples were tested for the presence of *A. capra* by nested PCR targeting the citrate synthase gene (*gltA*) as previously described [4, 8]. *Anaplasma* genus-specific primers were used for first round reaction, and primers specific for *A. capra* were used for nested reactions, which generated a product of 594 bp. In order to further characterize the *A. capra* strains isolated in the study, we amplified the partial sequences of the 16S rRNA gene (1,261 bp), the heat-shock protein gene (*groEL*, 874 bp), and the major surface protein 4 gene (*msp4*, 656 bp) from positive samples. The oligonucleotide primer sequences used in this study are shown in Table 1. PCR reactions were performed in an automatic thermocycler (Bio-Rad, Hercules, USA) as previously described [4, 8]. The DNA extracted from sheep infected with *A. capra* (GenBank accession no. KX417324) was used as the positive control, and sterile water was used as the negative control. Amplified fragments were subjected to electrophoresis on 1.0% agarose gel, staining with ethidium bromide (0.5 μg/ml), and visualized by UV transillumination.

**Table 1:** Primers and PCR amplification conditions

Target gene	Primer name	Primer sequence (5'-3')	Annealing temperature (°C)	Amplicon size (bp)	Reference
*gltA*	Outer-f	GCGATTTTAGAGTGYGGAGATTG	55	1031	[4]
	Outer-r	TACAATACCGGAGTAAAAGTCAA			
	Inner-f	TCATCTCCTGTTGCACGGTGCCC	60	594	[8]
	Inner-r	CTCTGAATGAACATGCCCACCCT			
16S rRNA	Forward	GCAAGTCGAACGGACCAAATCTGT	58	1261	[8]
	Reverse	CCACGATTACTAGCGATTCCGACTTC			
*groEL*	Forward	TGAAGAGCATCAAACCCGAAG	55	874	[8]
	Reverse	CTGCTCGTGATGCTATCGG			
*msp4*	Forward	GGGTTCTGATATGGCATCTTC	53	656	This study
	Reverse	GGGAAATGTCCTTATAGGATTCG			

### DNA sequencing and phylogenetic analysis

PCR products were purified with the TaKaRa Agarose Gel DNA purification Kit Ver. 2.0 (TaKaRa, Dalian, China), cloned into pGEM-T Easy vector (Promega, Madison, WI, USA) and transformed into *Escherichia coli* JM109 competent cells (TaKaRa, Dalian, China). Two recombinant clones were randomly selected for sequencing (Sangon Biotech, Shanghai, China). The obtained sequences were analyzed by a BLASTn search (http://blast.ncbi.nlm.nih.gov/Blast.cgi) in GenBank and by using the Clustal W method in the MegAlign software (DNAStar, Madison, WI). Phylogenetic trees were constructed based on the sequence distance method using the neighbor-joining (NJ) algorithm with the Kimura two-parameter model. Bootstrap values were determined by using 1,000 replications [9].

### Statistical analysis

Statistical analysis was conducted using a Chi-square test in Predictive for Analytics Software Statistics 18 (PASW, SPSS Inc., Chicago, IL, USA). *P*-values of 0.05 or less were considered statistically significant.

### Nucleotide sequence accession numbers

The sequences obtained in this study were deposited in the GenBank database under accession numbers as follows: KX417195–KX417207 for 16S rRNA, KX417308–KX417340 for *gltA*, KX417341–KX417356 for *groEL* and KX417357–KX417370 for *msp4*.

## Results

*Anaplasma capra* was identified in 75 of 547 animals, with an overall prevalence of 13.7% (Table 2). This organism was detected in 11 out of 24 study sites. The infection rates in the survey sites ranged from 0 to 78.6% (Table 2), and it varied considerably between the sampling locations. The infection rate of *A. capra* was slightly higher in sheep (16.3%) than in goats (12.3%) but the difference was not significant (*χ* ^*2*^ = 1.669, *df* = 1, *P* > 0.05).

**Table 2:** Detection of *A. capra* by PCR based on *gltA* gene in sheep and goats from China, 2011–2015

Location		Species	No. tested	Positive (%)
Province	County			
Chongqing	Wanzhou	Goat	24	4 (16.7)
	Jiangjin	Goat	30	2 (6.7)
Guangxi	Pingxiang	Goat	11	1 (9.1)
	Jingxi	Goat	19	0 (0)
Guizhou	Dushan	Goat	17	2 (11.8)
	Rongjiang	Goat	25	8 (32.0)
Hebei	Wangdu	Sheep	19	0 (0)
Hainan	Haikou	Goat	28	0 (0)
Sichuan	Hejiang	Goat	32	0 (0)
	Panzhihua	Goat	32	4 (12.5)
Shanxi	Lvliang	Sheep	50	0 (0)
Guangdong	Qingyuan	Goat	30	0 (0)
	Zhaoqing	Goat	33	1 (3.0)
Yunnan	Ruili	Goat	4	0 (0)
	Fuyuan	Goat	7	0 (0)
	Yanshan	Goat	15	0 (0)
Liaoning	Haicheng	Sheep	23	8 (34.8)
	Huanren	Goat	16	11 (68.8)
	Fengcheng	Goat	14	11 (78.6)
Inner Mongolia	Eerguna	Goat	20	0 (0)
	Manzhouli	Sheep	15	0 (0)
	Xinbaerhuzuoqi	Sheep	20	0 (0)
	Aershan	Sheep	18	0 (0)
Hubei	Suizhou	Sheep	45	23 (51.1)
Total			547	75 (13.7)

**Fig. 1:**
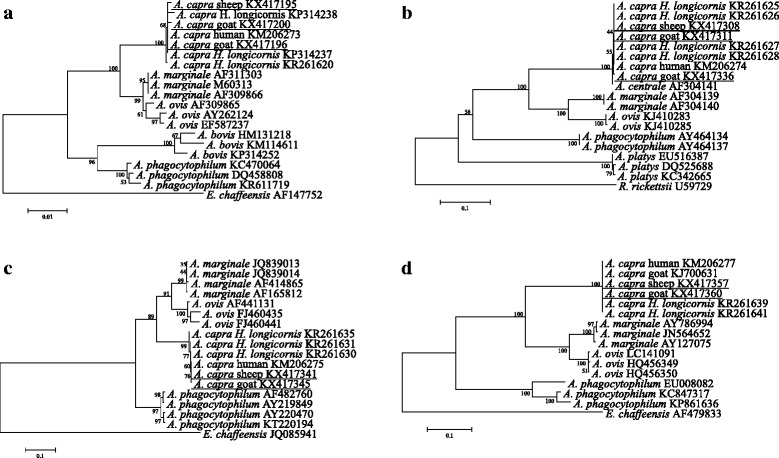
Phylogenetic analysis of “*Anaplasma capra*” and other members in the genus *Anaplasma* based on the 16S rRNA (**a**), *gltA* (**b**), *groEL* (**c**), and *msp4* (**d**) genes. Bootstraps analysis was performed with 1,000 replicates. Sequences obtained from this study are underlined. *Ehrlichia chaffeensis* and *Rickettsia rickettsii* were used as outgroups

The molecular characterization of *A. capra* isolates in sheep and goats was analyzed based on *gltA*, 16S rRNA, *groEL* and *msp4* genes. Thirty-three *gltA* sequences (594 bp, 23 from goats and 10 from sheep) of *A. capra* representative of different geographical locations were obtained in this study, and they provided two sequence variants that have 99.6–100% identity to the type strain HLJ-14 of *A. capra* detected in humans, goats and *Ixodes persulcatus* (GenBank KM206274) [4]. The 16S rRNA gene sequences (1,261 bp, 6 from goats and 7 from sheep) obtained from the *gltA* gene positive samples were 99.9–100% identical to each other and to strain HLJ-14 of *A. capra* (GenBank KM206273). Moreover, both the *groEL* (874 bp, 8 from goats and 8 from sheep) and *msp4* (656 bp, 8 from goats and 6 from sheep) gene sequences showed 100% similarity to the corresponding sequences of *A. capra* (GenBank KM206275 and KM206277). Phylogenetic analysis of 16S rRNA sequences showed that the isolates identified in this study were closely related to and clustered within the same clade with *A. capra* strain HLJ-14, but distinct from remaining recognized *Anaplasma* species (Fig. 1a), suggesting the novelty of this *Anaplasma* species. Similar phylogenetic organizations were inferred from the sequence analysis of *gltA*, *groEL* and *msp4* genes (Fig. 1b-d).

## Discussion

*Anaplasma* spp. are important tick-borne bacteria of veterinary and public health significance [10]. The genus *Anaplasma* encompasses six recognized species that infect various mammals and specific host cell types, such as monocytes, neutrophils, erythrocytes and platelets depending on the host species [11]. The order Rickettsiales was reorganized in 2001, and *Ehrlichia equi*, *Ehrlichia phagocytphilia* and the agent of human granulocytic ehrlichiosis were unified and named as *Anaplasma phagocytophilum* [11]*. Anaplasma phagocytophilum* has been known for almost a century to cause tick-borne fever (TBF) in ruminants, and more recently, it has been associated with human granulocytic anaplasmosis (HGA) [12]. In 2010, an *Anaplasma ovis* variant was detected in a patient in Cyprus [13]. Aside from the aforementioned species, no other causative agent of human anaplasmosis has been reported. However, the virulence of a novel *Anaplasma* species of genus *Anaplasma*, with the provisionally name *A. capra*, have recently been confirmed in China [4]. This species was first identified in goats, and shortly thereafter was recognized to be a new causative agent of human anaplasmosis [4]. In the present study, we did an active surveillance of *A. capra* in small ruminants in China, and 13.7% of 547 sheep and goats were naturally infected with this novel *Anaplasma* species. The prevalence of *A. capra* in goats and sheep differed among geographic regions, and it was identified in 11 of 24 investigated counties within 12 provinces in China. The findings of present study suggested that *A. capra* is widely distributed in China, and sheep and goats are the competent reservoir hosts for *A. capra*. The knowledge of the presence of *A. capra* in sheep and goats provides information for assessing the public health risks for human anaplasmosis.

Phylogenetic analysis of *A. capra* based on four conventional genetic markers (16S rRNA, *gltA*, *groEL* and *msp4* genes) strongly supported that the isolates obtained from goats, sheep, *Ixodes persulcatus* ticks and humans create a separate clade within the genus *Anaplasma*, suggesting that these *A. capra* strains possess the same molecular characteristics. In a previous report, a novel *Anaplasma* species closely related to *A. capra* has also been identified in *Haemaphysalis qinghaiensis* ticks in northwestern China [8]. The 16S rRNA gene of those isolates exhibit the highest sequence similarity with *A. capra* (similarity of 99.8–99.9%), but the *gltA* and *groEL* genes were relatively less identical to *A. capra* (88.6–88.7% for *gltA* and 90.6–91.0% for *groEL*). This organism has also been detected previously in deer (*Anaplasma* sp. NS104, GenBank AB454075) and in free living serows (*Anaplasma* sp. Kamoshika17, GenBank AB509223) in Japan [7]. Apparently, the *Anaplasma* species identified in those domestic and wild animals, ticks and humans should be a single species according to the criteria for classification of bacteria (at least 99% 16S rRNA gene homology) [14]. These findings indicated that *A. capra* may have high degree of genetic diversity and host tropisms, as have been confirmed in *A. phagocytophilum* [15]. There are at least two genotypes/genospecies of *A. capra* circulate in nature, one genotype contains strains isolated from goats, sheep, *I. persulcatus* and humans, while the other from deer, serows and *H. qinghaiensis*. Further studies should be conducted to clarify if these two *A. capra* genotypes have variation in pathogenicity.

It is well known that the members in the genus *Anaplasma* are transmitted transtadially rather than transovarially (from adult ticks to eggs) by ticks [10]. *Anaplasma capra* has been identified in *I. persulcatus* and *H. longicornis* ticks in China [4, 16]. However, pathogen detection in arthropods is not sufficient to validate its vector competence. Thus, their competency as vectors for *A. capra* remains to be proven, and wide tick surveillance should be conducted to determine the distribution and potential tick vectors of *A. capra*. To date, there is very little information of the natural cycles of *A. capra*, and the reservoir host is essential to keep *Anaplasma* organisms in nature [15]. As already mentioned, *A. capra* has been detected in goats, deer and serows [5–7], suggesting that this pathogen may affect a wide range of mammals, especially small ruminants. However, it is unclear at present whether *A. capra* can infect other mammalian species, and more research is needed to evaluate the full range of reservoir hosts for *A. capra*.

In addition, there is also significant difference in several biological features between *A. capra* and other well-recognized *Anaplasma* species. *Anaplasma* species organisms usually infect bone marrow-derived cells in blood, however, *A. capra* may infect endothelial cells in vivo [4, 17]. Therefore, microscopic examination of peripheral blood smears is not sensitive enough for diagnosis of *A. capra* infection in animals and humans. The symptoms caused by *Anaplasma* spp. are usually mild and nonspecific and can easily be confused with other infections [18]. These diseases would be generally neglected. However, increasing numbers of anaplasmosis cases of undetermined cause have been reported in mainland China and the USA [2, 19]. Clinicians and veterinary practitioners should pay more attention to this new emerging infectious disease in areas where anaplasmosis can occur.

## Conclusions

An active surveillance of *A. capra* was conducted in domestic small ruminants in China. Our survey showed that this novel *Anaplasna* species is widespread across China with an overall prevalence of 13.7% in goats and sheep. The *A. capra* isolates identified from animals, ticks and patients possess the same molecular characteristics, suggesting that this organism could be a substantial health threat to both animals and human beings.

## Abbreviations

DNA: Deoxyribonucleic acid
EDTA: Ethylene diamine tetraacetic acid
*gltA*: Citrate synthase
*groEL*: Heat shock operon
*msp*: Major surface protein
PCR: Polymerase chain reaction
UV: Ultraviolet
